# Advancing Our Understanding of the Excited States of the Tantalum Anion

**DOI:** 10.1021/acs.jpca.4c04032

**Published:** 2024-08-31

**Authors:** Maria Barysz

**Affiliations:** Faculty of Chemistry, Nicolaus Copernicus University in Toruń, Gagarina 7, 87-100 Toruń, Poland

## Abstract

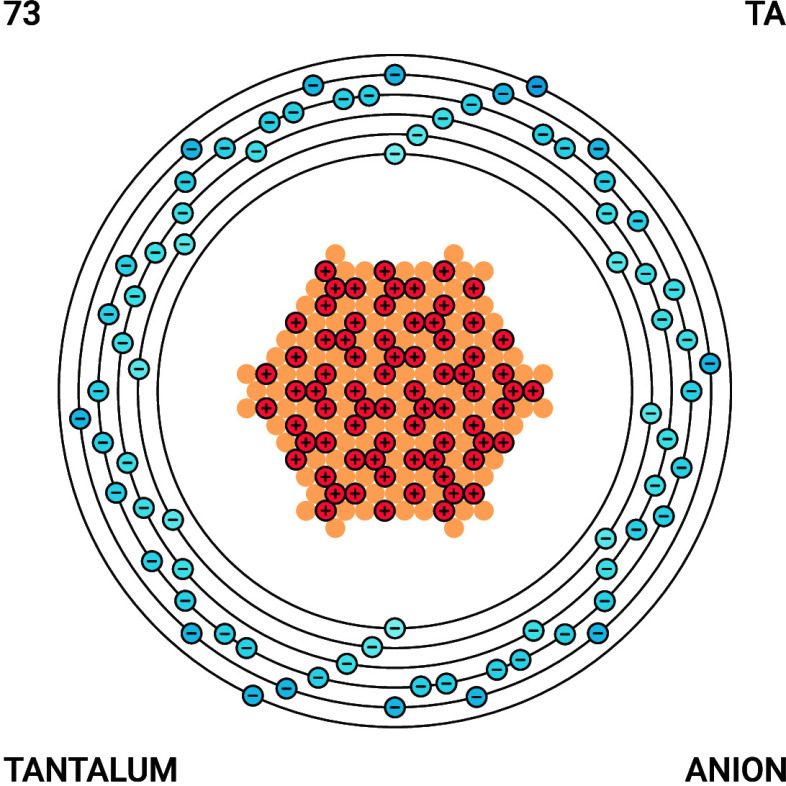

Our study provides a comprehensive theoretical examination of the energy levels associated with the neutral tantalum atom and its ions in various charge states (Ta, Ta^+^, and Ta^–^), employing the multiconfiguration Dirac–Hartree–Fock (MCDHF) method, and relativistic infinite order two-component (IOTC) method with multiconfiguration complete active space self-consistent field (CASSCF) followed by the second-order single-state multireference perturbation (CASPT2) methods. The effect of spin–orbit (SO) coupling is introduced via the restricted active space state interaction (RASSI) method, utilizing atomic mean field SO integrals (AMFI). Through IOTC CASSCF/CASPT2 RASSI calculations, we determined the electron affinity (EA) of the tantalum atom to be 0.321 eV, which stands among the most accurate theoretical values achieved to date. This result closely aligns with the experimental measurement of 0.329 eV. Our investigation highlights potential discrepancies between the predicted symmetry of the excited states of the tantalum anion and experimental observations. Additionally, we calculated the bonding energies for transitions from Ta^–^ to Ta and identified four potential bound or quasi-bound states in the tantalum anion.

## Introduction

Under typical chemical conditions, tantalum (Ta) primarily forms cations rather than anions. As a transition metal, tantalum, like most transition metals, tends to lose electrons to achieve stable electron configurations rather than gaining them to form anions. However, in specific environments or under certain conditions, such as in the gas phase or at high temperatures, tantalum can form anions and bound or quasi-bound (metastable) states.

These bound states are essential aspects of the electronic structure of anions and play a crucial role in understanding their properties and behavior. Generally, neutral atoms and positive ions can have an infinite number of bound states, while atomic anions typically have fewer bound states. This difference is one reason why atomic anions are particularly interesting to study.

Bound states in atomic anions are characterized by energy levels below the ionization threshold. The ionization threshold is the energy required to remove one or more electrons from the atom, resulting in a neutral atom or higher-charged ions. Bound states in atomic anions influence various chemical properties and behaviors of anions, including reactivity, chemical bonding, and spectroscopic properties. The energy levels of bound states influence the absorption and emission spectra of atomic anions and can be studied experimentally to understand their electronic structures. The tantalum anion possesses the most complex photoelectron spectrum among all atomic anions of transition elements, and this complexity has been a major obstacle in accurately measuring and studying its electron affinity and energy levels.

Measurements of tantalum’s electron affinity date back to 1981, when it was determined to be 0.323 eV.^1^ More recent, precise measurements utilizing the slow-electron velocity map imaging method combined with a cryogenic trap, have yielded a value of 0.328 eV.^2^ Additionally, three excited states, ^5^D_1_, ^3^P_0_, and ^5^D_2_ of Ta^–^ were observed as a result of these measurements.

Considering the difficulty of spectroscopic experimental studies, it is worthwhile to explore how theory and quantum mechanics describe the spectrum of the tantalum anion. From a theoretical standpoint, studying the tantalum atom also presents significant challenges. Being a heavy element, tantalum requires advanced theoretical methods capable of accounting for both correlation and relativistic effects simultaneously.

Before embarking on theoretical studies of the tantalum anion using specific methods, it is essential to assess the performance of these methods with the neutral tantalum atom and its cation. This s crucial due to the extensive amount of experimental data available for the neutral tantalum atom and its cation. The main goal of the presented work is to calculate the ground and excited states of the neutral tantalum atom and its cationic states, and to confirm or disprove the experimental observation of bound states of the tantalum anion. Additionally, we aim to interpret certain differences that arise between theoretical and experimental descriptions.

In the presented work, we will employ two methods: the multiconfiguration Dirac-Hartree–Fock (MCDHF) atomic method,^3,4^ and the spin-free version of the infinite order two-component (IOTC) method,^5−7^ combined with the multiconfiguration complete active space self-consistent field (CASSCF) molecular method,^8−10^ followed by the second-order single-state multireference perturbation (CASPT2) scheme.^11,12^ Within the IOTC CASCF/CASPT2 RASSI approach, the effect of spin–orbit (SO) coupling is introduced via the restricted active space state interaction (RASSI) method, utilizing atomic mean field SO integrals (modified AMFI).^13,14^

It is worth noting that for a considerable period, the relativistic second-order two-component Douglas-Kroll-Hess (DKH2) method,^15,16^ was among the most widely used approaches for calculating atomic and molecular relativistic effects. However, in the 2000s, new exact two-component methods began to emerge.^17−26^ One of these, the infinite-order two-component (IOTC) method, was formulated by the author.^5−7^ Our calculations will thus provide another test of the accuracy of the IOTC method.

## MCDHF AND IOTC METHODS

The four-component MCDHF calculations that have been used in the present work, were described in detail in refs^3, 4^. so that, hereafter, only the most important features of the MCDHF approach will be briefly reviewed. In the MCDHF method a system of N electrons is described by the relativistic Hamiltonian
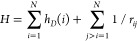
1where *h*_*D*_(*i*) is the one-electron Dirac operator for the *i*th electron:
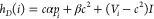
2In eq 2, *c* represents the velocity of light, α and β are the Dirac matrices, *p*_*i*_ is the electron momentum, *V*_*i*_ is the one-electron Coulomb potential (*V*_*i*_ = −*Z*/*r*_*i*_), and *I* is the unit matrix. The 1/*r*_*ij*_ describes the two-electron Coulomb interaction.

Relativistic corrections beyond the Dirac-Coulomb approximation for many-electron system are implemented using assumptions based on one-electron concept. For example, in the transverse photon interaction
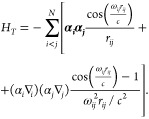
3which is the leading correction to the electron–electron Coulomb interaction, the frequency ω_*ij*_ is assumed to be the diagonal orbital energy parameters. This frequency is multiplied by a scale factor 10^–6^.^27^

The transverse photon interaction with the scaled frequencies is usually referred as the Breit interaction. The Breit interaction together with quantum electrodynamics QED corrections (self-energy and vacuum polarization) are added perturbatively after the MCDHF calculations.^28,29^

Compared to four-component MCDHF method the infinite-order two-component theory IOTC leads to enormous reduction of the computational effort and simultaneously recovers most of the relativistic effects which are accounted for within the Dirac formalism. This infinite-order two-component theory has been shown to completely recover the positive part of the Dirac spectrum for one electron systems.^5,7^ The method is based on the Fouldy–Wouthuysen idea and the separation of the electronic and positronic spectra of the four–component Dirac theory to the exact two-component form by the unitary transformation *U* of the one-electron Dirac Hamiltonian *h*_*D*_:
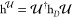
4with *h*_*D*_ defined by eq 2 and
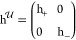
5

The unitary transformation *U*^†^*h*_*D*_*U* is based on the idea of Huelly et al. and is determined in terms of the auxiliary operator *R*.^30^

The infinite–order solution of the block-diagonalization problem is then reduced to the solution of the following operator equation:
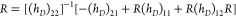
6

Once the solution *R* of eq 6 is known, the *exact* two-component “electronic” Hamiltonian *h*_+_ becomes
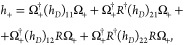
7where the Ω_+_ operator is defined through the *R* operator:.^5,7^
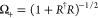
8

One of the possible ways to solve the eq 6 is by using an iterative scheme. This can be achieved through some odd powers of α, say α^2*k*–1^, *k* = 2,3,···(with α denoting the fine structure constant, α = 1/*c*). Consequently, the unitary transformation *U* will be exact up to the same order in α. Simultaneously, this approach will lead to an approximate form *h*_2*k*_, *k* = 2,3,···of *h*_+_. Thus, the method generates a series of two-component relativistic Hamiltonians whose accuracy depends on the accuracy of the iterative solution for *R*. In iteration step, the analytical forms of the *R* operator (eq eq 6) and the Hamiltonian *h*_+_ (eq 7) must be derived.

In a simplified manner, it can be said that this approach yields Douglas–Kroll–Hess Hamiltonians of order *n* (DKH_*n*_), *n* = 1,2,··· However, the original DKH_*n*_ Hamiltonians were derived differently.

In the two-component infinite-order IOTC method, the analytical form of the *R* operator equation is formulated only once, and the iterative procedure is defined within of the atomic/molecular code. The solution is exact within the given basis set. This is the main advantage of the IOTC method compared to the DKH_*n*_ methods.

## COMPUTATIONAL METHODOLOGY

In the IOTC CASSCF/CASPT2 RASSI methodology, all calculations are carried out using the complete active space self-consistent field (CASSCF) method,^8−10^ followed by multistate second-order multireference perturbation theory (CASPT2).^11,12^ The correct selection of the active space is crucial for the method and determines the accuracy of the calculations. Ideally, this space should be as large as possible; however, in practice, this is often not feasible. In the presented research, we employed two active spaces: the first comprised the valence 5d, 6s, and 6p atomic orbitals of tantalum, while the second, larger one included the valence 5d, 6s, 6p, 6d, and 7s atomic orbitals, resulting in nine and 15 active orbitals, respectively.

This part of the molecular calculations is carried out using *C*_2_ symmetry. The orbital subspaces (frozen/inactive/active) are defined by the number of orbitals in the irreducible representations of that group (A,B). The partition of the orbital space used in CASSCF calculations is then (0,0/15,19/6,3; *n*_*el*_) and (0,0/15,19/12,3; *n*_*el*_), where *n*_*el*_ is the number of electrons in the active space. For the neutral atom, cation, and negative ion of tantalum, *n*_*el*_ is equal to 5, 4, and 6, respectively.

State-average CASSCF calculations are performed. Six sextet states, ten quartet states, and nine doublet states are calculated for the tantalum atom. Twenty-six triplet states and ten quintet states are calculated for the tantalum cation, and nine quintet states are calculated for the tantalum anion.

The CASSCF wave functions and energies need to be further improved to account for the dynamic electron correlation contributions from subvalence shells. This is achieved by employing the CASPT2 method.

In the CASPT2 method, the treatment of electron correlation is expanded to include the 5s, 5p, and 4f electrons of Ta. These calculations are described by the (14, 9/1, 10/6, 3; *n*_*el*_) partition, with a frozen space of (14,9). Additionally, to highlight the importance of internal electrons, another partition, (14, 19/1, 0/6, 3; *n*_*el*_), is used in CASPT2 when the 5p and 4f orbitals are excluded from the correlation. This methodology is also applied to larger (12,3) active space.

All calculations conducted in the study of the tantalum atom and its ions are performed using Gaussian-type orbitals (GTO/CGTO). We use the large Gaussian basis set known as atomic natural orbital relativistic core correlating (ANO-RCC) (L-ANO-RCC): Ta [24s.21p.15d.11f.4g.2h/11s.10p.9d.8f.4g.2h].^31^

The philosophy of MCDHF calculations differs from that of the CASSCF/CASPT2 approach. First, we select active orbitals to construct the reference Configuration State Functions (CSFs) for the target state, referred to as spectroscopic orbitals. These are all optimized during the MCDHF procedure. Next, we introduce additional orbitals, known as correlation orbitals, to build CSFs that correct the reference CSFs by accounting for correlation effects. Optimizing all radial atomic, spectroscopic, and correlation orbitals simultaneously is usually not feasible. Instead, calculations are carried out using a layer-by-layer procedure. Initially, multireference calculations are performed where the orbitals are required to be spectroscopic. In subsequent steps, we systematically expand the active space by adding layers of correlation orbitals. Only the outermost layer of orbitals is optimized at each step, while the remaining orbitals are fixed from previous calculations.^27^

It differs from the CASSCF method, in which all internal and active orbitals are optimized in one step. The selection of spectroscopic orbitals depends on the system under investigation.

In the current MCDHF calculations, we consider all single and double excited (SD) configurations, as well as single, double, and triple excited (SDT) configurations. For the tantalum atom, these involve excitations of electrons from the 5d, 6s, 6p, 6d, and 7s orbitals.

## Results and Discussion

In Table 1, we present the excitation energies for 14 selected excited states of the tantalum atom, as determined by our MCDHF (and MCDHF + Breit correction) calculations. Additionally, we provide the symmetry of atomic levels, weights of dominant terms in the configuration state functions (CSFs), and electron configurations. These results are compared with experimental data^32^ for validation.

**Table 1:** Atomic Energy Levels of Tantalum Neutral Atom Obtained Using the SD and SDT MCDHF Method. All Data Are in eV^a^

Levels	Exp.^b^	MCDHF SD		MCDHF SDT		Dominant	Electron
		5d6s6p6d	5d6s6p6d7s	5d6s6p6d	5d6s6p6d7s	terms	configuration
1. ^4^F_3/2_	0.00	0.00(0.00)	0.00(0.00)	0.00	0.00	0.86 ^4^F	5d^3^6s^2^
2. ^4^F_5/2_	0.25	0.20(0.19)	0.20(0.19)	0.19	0.20	0.88 ^4^F	5d^3^6s^2^
3. ^4^F_7/2_	0.49	0.42(0.41)	0.42(0.41)	0.41	0.42	0.87 ^4^F	5d^3^6s^2^
4. ^4^F_9/2_	0.70	0.64(0.62)	0.64(0.62)	0.63	0.63	0.82 ^4^F	5d^3^6s^2^
5. ^4^P_1/2_	0.75	0.73(0.72)	0.73(0.73)	0.83	0.86	0.61 ^4^P	5d^3^6s^2^
						+0.18 ^4^P	5d^4^6s
6. ^4^P_3/2_	0.75	0.78(0.77)	0.79(0.78)	0.88	0.90	0.60 ^4^P	5d^3^6s^2^
						+0.16 ^4^P	5d^4^6s
7. ^4^P_5/2_	1.15	1.06(1.05)	1.07(1.06)	1.18	1.22	0.67 ^4^P	5d^3^6s^2^
						+0.18 ^4^P	5d^4^6s
8. ^2^G_7/2_	1.20	1.33(1.32)	1.36(1.35)	1.38	1.40	0.81 ^2^G	5d^3^6s^2^
9. ^6^D_1/2_	1.21	1.16(1.16)	1.12(1.11)	1.15	1.25	0.87 ^6^D	5d^4^6s
10 ^6^D_3/2_	1.24	1.20(1.20)	1.15(1.15)	1.19	1.29	0.90 ^6^D	5d^4^6s
11 ^2^G_9/2_	1.33	1.46(1.45)	1.51(1.49)	1.52	1.53	0.41 ^2^G + 0.23^2^H	5d^3^6s^2^
						+0.16 ^6^D	5d^4^6s
12 ^6^D_5/2_	1.39	1.29(1.28)	1.24(1.24)	1.29	1.39	0.89 ^6^D	5d^4^6s
13 ^6^D_7/2_	1.52	1.38(1.38)	1.33(1.33)	1.39	1.49	0.81 ^6^D	5d^4^6s
14 ^6^D_9/2_	1.66	1.52(1.51)	1.45(1.44)	1.49	1.62	0.83 ^6^D	5d^4^6s

a In brackets MCDHF + Breit correction.

b NIST Experiment. Reference (32).

We employed two sets of correlation orbitals: 5d6s6p6d and 5d6s6p6d7s, along with two versions of the MCDHF method: SD and SDT.

As shown in Table 1, the ground state of the tantalum atom is the ^4^F_3/2_ level. The subsequent three excited states are the ^4^F_5/2_, ^4^F_7/2_ and ^4^F_9/2_ levels, with their respective SD and SDT excitation energies showing good agreement with experimental data.

Following these, the next three levels are ^4^P_1/2_, ^4^P_3/2_ and ^4^P_5/2_. Both the calculated SD MCDHF excitation energies and the order of the levels exhibit good agreement with experimental findings. Interestingly, adding triple excitations does not improve the results; instead, it significantly alters the calculated energies. For ^4^P_1/2_ and ^4^P_3/2_, this alteration leads to a poorer correlation with experimental results.

A noticeable deviation, present in all models, occurs for the eighth and 11th states, ^2^G_7/2_, and ^2^G_9/2_, where the calculated excitation energy exceeds the experimental value by approximately 0.2 eV.

Our research shows that these are general problems with doublet states, which arise from difficulties in achieving energy convergence for these states. It was not possible to converge the energy for the ^2^P levels. The analysis of the experimental results for the doublet states in refs^32, 34^. also shows considerable discrepancies. These discrepancies are likely due to a significant contributions from more than one electronic state and configuration. Therefore, the ^2^P levels were omitted in Tables 1.

The calculation results for the next two states ^6^D_1/2_ and ^6^D_3/2_, demonstrate good agreement with the experimental data. The SD MCDHF results for the last three highest levels ^6^D_5/2_, ^6^D_7/2_, and ^6^D_9/2_, exhibit a larger discrepancy with the experimental data. Notably, incorporating triple excitations in the larger 5d6s6p6d7s active space aligns the calculated values closely with their experimental counterparts.

At the conclusion of this phase of the research, it is valuable to note that the dominant electronic configurations of the initial eight states and the state ^2^G_9/2_ are 5d^3^6s^2^, transitioning to configurations of 5d^4^6s for the subsequent states. However, we can notice that the influence of the 5d^4^6s configuration notably affects the ^4^P and ^2^G_9/2_ states.

Furthermore, as highlighted in Table 1, the Breit corrections to individual states exhibit varying magnitudes, ranging from 0.00 to −0.02 eV. Quantum electrodynamics (QED) corrections, including self-energy (SE) and vacuum polarization (VP), are negligible in size, and fall within the precision of our current work.

In Table 2, we present a similar analysis for the positively charged tantalum ion. Previous theoretical ab initio results for this ion are not available. The symbols *a* and *b* are used to distinguish between the lower and higher ^3^F excited levels, respectively.

**Table 2:** Atomic Energy Levels of Tantalum Cation Obtained Using the SD and SDT MCDHF Method. All Data Are in eV

Levels	Exp.^a^	SD MCDHF		SDT MCDHF		Dominant	Electron
		5d6s6p6d	5d6s6p6d7s	5d6s6p6d	5d6s6p6d7s	terms	configuration
1. ^5^F_1_	0.00	0.00	0.00	0.00	0.00	0.96 ^5^F	5d^3^6s
2. ^5^F_2_	0.13	0.11	0.11	0.11	0.11	0.96 ^5^F	5d^3^6s
3. ^5^F_3_	0.33	0.27	0.27	0.27	0.28	0.98 ^5^F	5d^3^6s
4.a^3^F_2_	0.39	0.52	0.56	0.36	0.33	0.67 ^3^F	5d^2^6s^2^
5. ^3^P_0_	0.51	0.53	0.54	0.57	0.55	0.39 ^3^P	5d^3^6s
						+ 0.30 ^3^P	5d^2^6s^2^
6. ^5^F_4_	0.55	0.46	0.46	0.47	0.47	0.97 ^5^F	5d^3^6s
7. ^3^P_1_	0.66	0.67	0.67	0.70	0.67	0.43 ^3^P	5d^3^6s
						0.30 ^3^P	5d^2^6s^2^
8. ^3^P_2_	0.70	0.77	0.77	0.76	0.73	0.34 ^3^P	5d^3^6s
						0.22 ^3^P + 0.11 ^3^F	5d^2^6s^2^
9. ^5^F_5_	0.77	0.67	0.67	0.68	0.68	0.95 ^5^F	5d^3^6s
10a^3^F_3_	0.85	0.93	0.97	0.77	0.74	0.78 ^3^F	5d^2^6s^2^
11a^3^F_4_	1.21	1.28	1.31	1.14	1.12	0.64 ^3^F	5d^2^6s^2^
12b^3^F_2_	1.20	1.29	1.26	1.30	1.25	0.47 ^3^F	5d^3^6s

a NIST Experiment. Reference (32).

The data presented in Table 2 demonstrate that the active space 5d6s6p6d is effectively saturated. Adding an additional 7s orbital no longer affects the energy values of the levels. While the inclusion of triple excitations is generally unnecessary for most calculated states, exceptions exist for the a^3^F_2_ and a^3^F_3_ states. For instance, in the case of the 5d6s6p6d7s active space and a^3^F_2_ state, a significant alteration in energy is observed upon the inclusion of triple excitations, decreasing from approximately 0.56 to 0.33 eV, thereby closely aligning with the experimental value of 0.39 eV. Similarly, for the a^3^F_3_ state, the energy shifts from 0.97 eV in the SD MCDHF approach to 0.74 eV in the SDT MCDHF method. The experimental value for this state is recorded as 0.85 eV (refer to Table 2).

In summary, the calculated excitation energies of the tantalum cation closely match the experimental values.

Table 3 presents the results of MCDHF calculations for the ground state and four excited states of the tantalum anion, along with the most recent experimental data.

**Table 3:** Atomic Energy Levels of Tantalum Anion Obtained Using the MCDHF Method. All Data Are in eV

Levels^a^	Exp.^a^	Levels^b^	MCDHF		Dominant	Electron
			5d6s6p	4f5d6s6p	terms	configuration
1. ^5^D_0_	0.000	^5^D_0_	0.00	0.00	0.86 ^5^D	5d^4^6s^2^
2. ^5^D_1_	0.13	^5^D_1_	0.06	0.07	0.88 ^5^D	5d^4^6s^2^
3. ^3^P_0_	0.22	^5^D_2_	0.16	0.18	0.90 ^5^D	5d^4^6s^2^
4. ^5^D_2_	0.28	^5^D_3_	0.29	0.30	0.89 ^5^D	5d^4^6s^2^
5.	-	^5^D_4_	0.43	0.44	0.87 ^5^D	5d^4^6s^2^

a Experiment. Reference (2).

b The current MCDHF assignment of symmetry.

As previously mentioned, this case is particularly intriguing for study. We have newly acquired experimental data, which were challenging to obtain,^2^ and there is a notable absence of corresponding theoretical findings.

Performing MCDHF calculations for the tantalum anion is more challenging than for the neutral atom or its cation due to numerous convergence issues. Because of these challenges, the active space for the anion could not be too large, so we chose the smaller 5d6s6p active space. It is sometimes believed that, in the case of anions, the internal orbitals are more significant than the valence orbitals. To verify this, we added 4f orbitals to the active space, resulting in the 4f5d6s6p configuration.

The results offer intriguing insights. Initially, the computed excitation energies show a high degree of agreement with the experimental values.

Second, notable differences arise in the assignment of symmetry to the electronic states of the anions. While both the ground state ^5^D_0_ and the first excited state ^5^D_1_ exhibit consistent symmetry in both computational and experimental findings, subsequent excited states show different symmetries.

Consequently, the second experimentally observed excited state corresponds to the ^3^P_0_ state, whereas calculations indicate the ^5^D_2_ state. Similarly, the third observed excited state is ^5^D_2_, while the calculations suggest the ^5^D_3_ level.

The theoretical calculations suggest that there is no ^3^P_0_ excited state at such low energy. The nearest computed ^3^P_0_ state has an excitation energy of 1.2 eV.

The inclusion of 4f internal orbital in the active space appears to be practically irrelevant for the calculated energies. We will continue to investigate this issue.

In summary, despite the good agreement between the calculated and experimental energies of the levels, the results of MCDHF calculations reveal some notable discrepancies. These findings necessitate the next phase of our investigation, which will involve employing a more sophisticated computational approach.

In the second stage of our research, we performed calculations using the IOTC CASSCF/CASPT2 RASSI method.

In Table 4, we present the tantalum excitation energies obtained from IOTC CASSCF/CASPT2 RASSI calculations in both small (6,3) and large (12,3) active spaces, with various combinations of frozen spaces, specifically (14,19) and (14,9). This approach allows us to investigate how correlation effects from the internal 5p and 4f orbitals influence the calculated excitation energies. We compare these results with experimental excitation energy published by NIST^32^ and with available theoretical research.^31,33^

**Table 4:** IOTC CASSF/CASPT2 RASSI Atomic Energy Levels of the Tantalum Neutral Atom. All Data Are in eV^a^

	Levels	Exp.^b^	IOTC	IOTC	IOTC	IOTC	DKH2^c^	DKH2^d^
			Act.(6,3)	Act.(12,3)	Act.(6,3)	Act.(12,3)	Act.(6,3)	Act.(6,3)
			Fr.(14,9)	Fr.(14,9)	Fr.(14,19)	Fr.(14,19)		
1.	^4^F_3/2_	0.00	0.00	0.00	0.00	0.00	0.00	0.00
2.	^4^F_5/2_	0.25	0.22	0.23	0.22	0.23	0.27	0.27
3.	^4^F_7/2_	0.49	0.46	0.48	0.46	0.48	0.54	0.54
4.	^4^F_9/2_	0.70	0.70	0.73	0.70	0.73	0.74	0.75
5.	^4^P_1/2_	0.75	0.75	0.72	0.84	0.80	0.75	0.72
6.	^4^P_3/2_	0.75	0.89	0.88	0.99	0.97	0.75	0.68
7.	^4^P_5/2_	1.15	1.27	1.15	1.24	1.24	1.20	1.18
8.	^2^G_7/2_	1.20	1.19	1.18	1.19	1.18	1.26	1.13
9.	^6^D_1/2_	1.21	1.06	1.10	1.20	1.22	1.11	1.41
10.	^6^D_3/2_	1.24	1.14	1.18	1.27	1.29	1.15	1.44
11.	^2^G_9/2_	1.33	1.69	1.71	1.66	1.69	1.39	-
12.	^6^D_5/2_	1.39	1.12	1.31	1.40	1.42	1.32	1.53
13.	^6^S_5/2_	1.46	3.80	4.01	3.93	4.00	-	-
14.	^6^D_7/2_	1.52	1.34	1.43	1.51	1.56	1.43	-
15.	^6^D_9/2_	1.66	1.56	1.65	1.74	1.80	1.60	-

a Configurations: 4F, ^4^P, 2G - 5d36s2; 6D, - 5d46s; 6S - 5d5.

b NIST Experiment. Reference (32).

c DKH2. Reference (31)

d DKH2. Unpublished results.^33^

The initial observation reveals a notably high IOTC CASSCF/CASPT2 RASSI excitation energy for the ^6^S_5/2_ state, approximately 4.0 eV. In contrast, the NIST data lists this value as 1.46 eV. Additionally, our MCDHF and cited DKH2^31,33^ calculation results, show that this state is absent from the calculated range of spectra.

As it turns out, this issue was resolved some time ago. The experimental results of B. Arcimowicz et al.^34^ from 2013 identify the presence of the ^6^S_5/2_ state with an excitation energy of 4.03 eV, which is consistent with our IOTC CASSCF/CASPT2 RASSI calculations.

The excitation energies of the lowest three excited levels ^4^F_5/2_, ^4^F_7/2_ and ^4^F_9/2_ do not depend on the size of the active space or the presence of the internal 5p and 4f orbitals in the correlation space of the CASPT2 method.

Including the 5p and 4f orbitals in the correlation significantly improves the accuracy of the calculated energy for the ^4^P_1/2_ level and substantially reduces the energy of the ^4^P_3/2_ level. However, the energy of the ^4^P_3/2_ level remains slightly overestimated relative to the experimental value. The SDT MCDHF method yields results that are comparable to the IOTC CASSCF/CASPT2 RASSI values (see Table 1).

The excitation energy of the ^4^P_5/2_ level is most accurately predicted using the large active space (12,3) and frozen space (14,9).

Starting from the excited ^6^D_1/2_ state, it is crucial to exclude internal orbitals from the correlation to accurately reproduce experimental results. Interestingly, the size of the active space appears to have minimal impact on these states.

The energy of the ^2^G_9/2_ level is poorly reproduced across all modeled spaces. Similar to the MCDHF calculations, it was not possible to achieve convergence for the ^2^P level energies. Based on the previously presented arguments, these levels have been omitted from Table 4.

Finally, it is worth noting that the earlier DKH2 calculation results cited in Table 4,^31,33^ particularly in the last two columns, show significant differences for the higher excited states of tantalum. The details of these calculations are not provided, but these differences are most likely due to convergence issues in the calculations from Reference.^33^

Our calculations thus far indicate that, apart from a single exception, a small active space is generally sufficient for obtaining satisfactory results. Therefore, in Table 5, we present the results for the tantalum cation using a small active space (6,3) in two scenarios: alongside frozen spaces (14,9) and (14,19).

**Table 5:** Tantalum Cation, IOTC CASSCF/CASPT2 RASSI Energy Levels. All Data Are in eV

	Levels	Exp.^a^		IOTC	IOTC
				Act.(6,3)	Act.(6,3)
				Fr.(14,9)	Fr.(14,19)
1.	^5^F_1_	0.00		0.00	0.00
2.	^5^F_2_	0.13		0.14	0.13
3.	^5^F_3_	0.33		0.34	0.34
4.	a^3^F_2_	0.39		0.49	0.36
5.	^3^P_0_	0.51		0.64	0.63
6.	^5^F_4_	0.55		0.59	0.58
7.	^3^P_1_	0.66		0.69	0.68
8.	^3^P_2_	0.70		0.82	0.76
9.	^5^F_5_	0.77		0.85	0.84
10.	a^3^F_3_	0.85		1.08	0.92
10.	a^3^F_4_	1.21		1.48	1.35
11.	b^3^F_2_	1.20		1.23	1.13

a NIST Experiment. Reference (32).

The results presented in Table 5 align well with the experimental data. However, for the a^3^F and ^3^P_2_ states, it is more advantageous to freeze a larger number of internal orbitals when using the CASPT2 method.

In brief, our calculations using the IOTC CASSCF/CASPT2 RASSI method show satisfactory agreement with experimental data for both the tantalum atom and its cation. The size of the frozen space in CASPT2 calculations is very important.

It is worth noting that these results represent an average of multiple CASSCF outcomes, where the procedure for the simultaneous convergence of energies from various states slightly affects the precision of the final results. Nevertheless, these findings suggest that calculations for the tantalum anion can be performed with high reliability.

The study of the tantalum anion presents significant challenges both experimentally and theoretically. Determining the electron affinity (EA) and the energies of excited states is particularly demanding. The latest measurement by Sheng Li et al.,^2^ which reports an electron affinity of 0.328858(23) eV, is now the recommended benchmark.

The theoretical electron affinity (EA) value, obtained using the large active space (12,3) and incorporating 5p and 4f correlation within the IOTC CASSCF/CASPT2 RASSI approximation, is 0.321 eV. This value is close to the calculated DKH2 EA value of 0.23 eV,^31^ and aligns remarkably well with the experimental value of 0.329 eV.

In Table 6, we present the IOTC CASSCF/CASPT2 RASSI ab initio theoretical results for the tantalum anion. This table displays two sets of calculations: one employing a small active space and another employing a large active space, both incorporating correlated 5p and 4f orbitals. Turning off the correlation of internal orbitals, specifically 5p and 4f, does not affect the energy values of the anion levels at all, so we do not show them.

**Table 6:** Tantalum Anion, IOTC CASSCF/CASPT2 RASSI Energy Levels. All Data Are in eV

	Levels^a^		Exp.^a^	Levels^b^	IOTC	IOTC
					Act.(6,3)	Act.(12,3)
					Fr.(14,9)	Fr.(14,9)
1.	^5^D_0_		0.00	^5^D_0_	0.00	0.00
2.	^5^D_1_		0.13	^5^D_1_	0.06	0.06
3.	^3^P_0_		0.22	^5^D_2_	0.17	0.18
4.	^5^D_2_		0.28	^5^D_3_	0.34	0.36
5.			-	^5^D_4_	0.56	0.59

a Experiment. Reference (2).

b The current IOTC CASSCF/CASPT2 RASSI assignment of symmetry.

Generally, the energy levels of the tantalum anion appear to be unaffected by the size of active space and the number of frozen orbitals.

The calculated excitation energies exhibit a strong correlation with the experimental values; however, the level symmetry do not. The first two calculated states are ^5^D_0_ and ^5^D_1_, which are in full agreement with the experimental data. However, the third and fourth excited states are ^5^D_2_ and ^5^D_3_, deviating from the experimentally observed ^3^P_0_ and ^5^D_2_. These findings are consistent with those obtained previously using the MCDHF method (see Table 3).

The obtained results suggest that the experimental assignment of symmetries to the studied states should be revisited.

Recently, Sheng Li et al.^2^ conducted measurements on the electron affinity of the tantalum anion, as well as on the energies associated with transitions from various states of the tantalum anion (Ta^1–^) to those of the neutral atom (Ta). These measurements are pivotal as they facilitate the determination of potential bound or quasi-bound (or metastable) states of the tantalum anion.

In Tables 7, 8, and 9, we present selected transition energies from Ta^1–^ to Ta using the IOTC CASSCF/CASPT2 RASSI and MCDHF methods. The binding energies depicted correspond to transitions from the ^5^D levels of the anion (the current assignment of symmetres is used in all tables) to the ^4^F, ^4^P, ^2^G, and ^6^D levels of the neutral atom.

**Table 7:** Photodetachment from Ta^–1^ to Ta Transitions Obtained Using the IOTC CASSCF/CASPT2 RASSI and MCDHF Methods. All Results Are in eV

Transition^d^^,^^e^	Energy		
Ta ← Ta^–1^	IOTC^a^	MCDHF^b^	Exp.^c^
1. ^4^F_3/2_ ←^5^D_3_(^5^D_2_)	–0.04	0.03	0.05
2. ^4^F_3/2_ ←^5^D_2_(^3^P_0_)	0.14	0.15	0.11
3. ^4^F_3/2_ ←^5^D_1_(^5^D_1_)	0.26	0.26	0.18
4. ^4^F_3/2_ ←^5^D_0_(^5^D_0_)	0.32	0.33	0.33
5. ^4^F_5/2_ ←^5^D_3_(^5^D_2_)	0.19	0.23	-
6. ^4^F_5/2_ ←^5^D_2_(^3^P_0_)	0.37	0.35	-
7. ^4^F_5/2_ ←^5^D_1_(^5^D_1_)	0.49	0.46	0.43
8. ^4^F_5/2_ ←^5^D_0_(^5^D_0_)	0.55	0.53	0.58
9. ^4^F_7/2_ ←^5^D_3_(^5^D_2_)	0.44	0.45	-
10.^4^F_7/2_ ←^5^D_2_(^3^P_0_)	0.62	0.57	-
11.^4^F_7/2_ ←^5^D_1_(^5^D_1_)	0.74	0.68	0.68
1. ^2^F_7/2_ ←^5^D_0_(^5^D_0_)	0.80	0.75	-
2. ^4^F_9/2_ ←^5^D_3_(^5^D_2_)	0.69	0.67	-
3. ^4^F_9/2_ ←^5^D_2_(^3^P_0_)	0.87	0.79	-
4. ^4^F_9/2_ ←^5^D_1_(^5^D_1_)	0.99	0.90	-
5. ^4^F_9/2_ ←^5^D_0_(^5^D_0_)	1.05	0.97	-

a IOTC CASSCF/CASPT2 RASSI: Act(12,3), Fr(14,9). Transitions are calculated with the calculated value of EA = 0.321 eV.

b MCDHF: Values are calculated with the experimental value of EA = 0.329 eV.

c Experimental results. See ref (2).

d The current assignment of symmetry for the anion.

e The corresponding experimental assignment of symmetry^2^ is given in parentheses.

**Table 8:** Photodetachment from Ta^–1^ to Ta Transitions Obtained Using the IOTC CASSCF/CASPT2 RASSI and MCDHF Methods. All Results Are in eV

Transition^d^	Energy		
Ta ← Ta^–1^	IOTC^a^	MCDHF^b^	Exp.^c^
1. ^4^P_1/2_ ←^5^D_3_	0.68	0.76	-
2. ^4^P_1/2_ ←^5^D_2_	0.86	0.88	-
3. ^4^P_1/2_ ←^5^D_1_	0.98	0.99	-
4. ^4^P_1/2_ ←^5^D_0_	1.05	1.06	-
5. ^4^P_3/2_ ←^5^D_3_	0.84	0.82	-
6. ^4^P_3/2_ ←^5^D_2_	1.02	0.94	-
7. ^4^P_3/2_ ←^5^D_1_	1.14	1.05	-
8. ^4^P_3/2_ ←^5^D_0_	1.20	1.12	1.08
9. ^4^P_5/2_ ←^5^D_3_	1.11	1.10	1.15
10.^4^P_5/2_ ←^5^D_2_	1.29	1.22	-
11.^4^P_5/2_ ←^5^D_1_	1.31	1.33	1.33
1. ^4^P_5/2_ ←^5^D_0_	1.47	1.40	1.45

a IOTC CASSCF/CASPT2 RASSI: Act(12,3), Fr(14,9). Transitions are calculated with the calculated value of EA = 0.321 eV.

b MCDHF: Values are calculated with the experimental value of EA = 0.329 eV.

c Experimental results. See ref.^2^

d The current assignment of symmetry for the anion.

**Table 9:** Photodetachment from Ta^–1^ to Ta Transitions Obtained Using the IOTC CASSCF/CASPT2 RASSI and MCDHF Methods. All Results Are in eV

Transition^d^	Energy		
Ta ← Ta^–1^	IOTC^a^	MCDHF^b^	Exp.^c^
1. ^2^G_7/2_ ←^5^D_3_	1.14	-	-
2. ^2^G_7/2_ ←^5^D_2_	1.32	-	-
3. ^2^G_7/2_ ←^5^D_1_	1.44	-	1.39
4. ^2^G_7/2_ ←^5^D_0_	1.50	-	1.53
5. ^6^D_1/2_ ←^5^D_3_	1.06(1.18)	1.15	-
6. ^6^D_1/2_ ←^5^D_2_	1.24(1.36)	1.27	-
7. ^6^D_1/2_ ←^5^D_1_	1.36(1.48)	1.38	1.39
8. ^6^D_1/2_ ←^5^D_0_	1.42(1.54)	1.45	1.54
9. ^6^D_3/2_ ←^5^D_3_	1.14(1.25)	1.18	1.28
10.^6^D_3/2_ ←^5^D_2_	1.32(1.43)	1.30	-
11.^6^D_3/2_ ←^5^D_1_	1.44(1.55)	1.41	1.42
1. ^6^D_3/2_ ←^5^D_0_	1.50(1.61)	1.48	1.57

a IOTC CASSCF/CASPT2 RASSI: Act(12,3), Fr(14,9). In brackets Act(12,3), Fr(14,19). Transitions are calculated with the calculated value of EA = 0.321 eV.

b MCDHF. Values are calculated with the experimental value of EA = 0.329 eV.

c Experimental results. See ref (2).

d The current assignment of symmetry for the anion.

For the IOTC CASSCF/CASPT2 RASSI method we used the calculated electron affinity of 0.321 eV, while the MCDHF calculations employed the experimental value of 0.329 eV.

The calculated energy values are then compared with those measured by Sheng Li et al.^2^

The results show a high degree of agreement, indicating that the experimental and calculated energies are in strong alignment.

In addition to the ground state ^5^D_0_ of the anion, the IOTC CASSCF/CASPT2 RASSI calculations reveal two additional quasi-bound (metastable) excited states for the tantalum anion: ^5^D_1_ and ^5^D_2_. Remarkably, the third excited state, ^5^D_3_, which is potentially quasi-bound, is positioned slightly higher, approximately 0.04 eV above the ground state ^4^F_3/2_ of the neutral atom.

Both the MCDHF calculations and experimental results suggest the existence of three quasi-bound (metastable) excited states. In particular, the ^5^D_3_ state approaches the ^4^F_3/2_ level from below by 0.03 eV in the MCDHF results and 0.05 eV in the experimental results, indicating that it could indeed be a quasi-bound state of the anion. Consequently, drawing definitive conclusions presents a challenge.

## Conclusions

We have provided a comprehensive and precise theoretical description of the energy levels of the neutral tantalum atom, as well as its positive, and negative ions. The computed IOTC CASSCF/CASPT2 RASSI electronic affinity of tantalum atom stands at 0.321 eV, making it one of the most accurate theoretical values obtained thus far.

Our research indicates that the symmetry of the third and fourth excited states of the tantalum anion, ^5^D_2_ and ^5^D_3_ respectively, may differ from the experimental predictions, which suggest ^3^P_0_ and ^5^D_2_, respectively.

These findings suggest that the experimental assignment of symmetries to the studied states should be revisited.

The MCDHF and IOTC CASSCF/CASPT2 RASSI calculations, along with experimental results, suggest the existence of two or three quasi-bound (metastable) excited states.

The calculated excitation energies for the tantalum atom, its cation, and anion, as well as the transition energies from Ta^1–^ to Ta, exhibit strong agreement with experimental values.

Finally, our study highlights the effectiveness of our relativistic two-component method (IOTC) and the high accuracy of the resulting data.

## Dedication

This work is dedicated to Professor Rodney Bartlett, a distinguished gure in Theo- retical Chemistry, on the occasion of his 80th birthday.
